# Management of chickpea Ascochyta blight using fungicides and cultivar resistance improves grain yield, quality, and grower profitability

**DOI:** 10.3389/fpls.2022.942220

**Published:** 2022-10-03

**Authors:** Joshua Fanning, Jason Brand, Isabel Munoz Santa, Linda McDonald, Julian Taylor, Grant Hollaway

**Affiliations:** ^1^Agriculture Victoria, Horsham, VIC, Australia; ^2^School of Agriculture, Food, and Wine, University of Adelaide, Glen Osmond, SA, Australia; ^3^Department of Statistics and Operations Research, University of Valencia, Valencia, Spain

**Keywords:** Ascochyta, chickpea, fungicides, grain yield loss, economics

## Abstract

International production of chickpea is under constant threat from the fungal disease Ascochyta blight (*Ascochyta rabiei*). In Australia, there is limited cultivar resistance, and disease management is reliant on foliar applied fungicides. Several recently registered fungicides in Australia that combine active ingredients with different modes of actions, have been shown to have curative properties. In this study, in the presence of Ascochyta blight, disease severity, grain yield and quality were measured and the subsequent gross margin for growers calculated in seven field experiments conducted in Victoria (Australia) across three seasons. These experiments investigated the effects of: two cultivars with differing disease resistance (PBA Striker and Genesis 090), and several fungicide strategies for the control of Ascochyta blight. Fungicides that combine different modes of actions (Tebuconazole + Azoxystrobin, Bixafen + Prothioconazole and Fludioxonil + Pydiflumetofen) were applied before a rainfall event (preventative) or after the first signs of disease (post-infection). Older, single active fungicides compared included Captan, Chlorothalonil, and Propiconazole, all applied preventatively. Maximum disease severities ranged from 87% at Horsham and 94% at Curyo across three seasons with Nhill recording 87% during 2020. Demonstrating the benefit of cultivar resistance for Ascochyta blight management, grain yield losses were substantially lower in the partially resistant cultivar Genesis 090 (64%) compared to the susceptible cultivar PBA Striker (96%), at Curyo in 2020. The preventative fungicide strategies reduced grain yield losses from 96 and 64% to 51 and 15% for PBA Striker and Genesis 090, respectively, demonstrating the benefit of fungicides in Ascochyta blight management. Across seasons and environments, a comparison between fungicides applied preventatively or post-infection highlighted both were both profitable ($23–$1,095/ha), except when dry conditions limited grain yield to less than 0.6 t/ha. The post infection timing had greater yield losses in sites/seasons with higher rainfall, but with dual active ingredient fungicides and partially resistant cultivars this timing could allow a reduction in the number of fungicide applications, thus improving profitability. These experiments highlighted the importance of controlling Ascochyta blight through cultivar resistance and fungicides to improve grain yields, grain quality, and grower profitability.

## Introduction

International production of chickpea (*Cicer arietinum* L.), a grain legume, is under constant threat from the fungal disease Ascochyta blight (AB) caused by the pathogen *Ascochyta rabiei* (teleomorph: *Didymella rabiei*; Davidson and Kimber, 2007; Banniza et al., 2011; Kukreja et al., 2018; Benzohra et al., 2020). Ascochyta blight can cause significant yield losses and complete crop failure in susceptible cultivars in the absence of control measures (Chongo et al., 2003; Bretag et al., 2008). To prevent grain yield losses due to AB, integrated disease management is recommended worldwide. This includes utilising partially resistant cultivars, avoiding inoculum sources through crop rotation, manipulating sowing times, sowing clean seed, and applying fungicides (Davidson and Kimber, 2007; Kukreja et al., 2018).

Currently there is limited genetic resistance available to suppress AB with researchers internationally searching for genetic resistance (Gayacharan et al., 2020; Newman et al., 2021). In the absence of adequate cultivar resistance, fungicides are required to control AB (Bretag et al., 2008). Previous research has focused on the use of fungicides, such as Chlorothalonil (Fungicide Group M5), Mancozeb (Group M3), Pyraclostrobin (Group 11), Azoxystrobin (Group 11), and Boscalid (Group 7; Chongo et al., 2003; Bretag et al., 2008; Banniza et al., 2011; Crutcher et al., 2022). However, in Australia, there have been several fungicide actives or mixes recently registered. These include Tebuconazole + Azoxystrobin (Fungicide Groups 3 and 11, Veritas®, Adama, Australia), Bixafen + Prothioconazole (Groups 7 and 3, Aviator®Xpro®, Bayer Crop Science, Australia), during 2016, and Fludioxonil + Pydiflumetofen (Groups 7 and 12, Miravis® Star, Syngenta Australia) during 2021 (Australian Pesticides and Veterinary Medicines Authority, 2022). Several of these fungicides advertise curative properties and Fludioxonil has been shown to have curative properties when controlling sclerotinia in oilseed rape (Duan et al., 2013).

The repeated application of fungicides during a season combined with a limited range of registered products in chickpeas increases the chance of fungicide resistance developing in the pathogen populations (Corkley et al., 2022). In North America, resistance of *Ascochyta rabiei* to prothioconazole and thiabendazole has been reported (Wise et al., 2009) and in Canada, Azoxystrobin and Pyraclostrobin resistance (Gossen and Anderson, 2004; Ahmed et al., 2014). Therefore, growers need access to effective fungicide strategies using a range of fungicides to allow rotation between actives and modes of action to slow or prevent resistance development. In Southern Australia, chickpeas are grown in winter-dominant rainfall areas and reliant on in season rainfall, with some areas receiving variable (Bretag et al., 2008). In some seasons, in these low rainfall environments, AB may not develop, yet some growers are still prophylactically applying fungicides with the industry recommendation prior to rain events. Some of the recently registered actives with curative properties may allow for a reduction in the number of fungicide applications in a season, by applying the fungicide when disease is present.

The main objective of this study was to evaluate the effect of cultivar resistance and several fungicide strategies (including recently registered fungicides with dual modes of action) on AB disease severity, grain yield, grain quality, and gross margins. Dual active fungicides were applied under two timing approaches, which included comparing the efficacy of a traditional preventative fungicide approach whereby fungicides are applied before rainfall events, compared a post-infection (reactive) approach, waiting for disease to be present before applying the fungicides with multiple modes of action. Experiments occurred, during 2018 to 2020, with two cultivars of differing resistance were compared, in three locations across Victoria (Australia).

## Materials and methods

### Experimental sites

Seven experiments were conducted in farmers’ paddocks across three sites during 2018 to 2020 in the key Victorian chickpea medium and low rainfall production zones (Table 1). Experiments were sown inter-row between the rows of cereal stubble retained from the previous crop. The experiments were planted during May (late autumn) of each year for a target establishment density of 35 plants/m^2^, with actual grain sown adjusted for germination and seed weight.

**Table 1:** Summary of experimental location and environmental data.

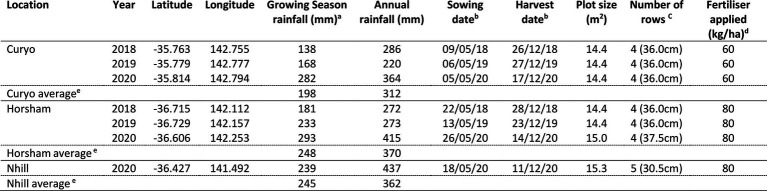

Rainfall data were obtained from the nearest meteorological stations at Curyo (Marbeled 77,028), Horsham (Horsham Aerodrome 79,100), and Nhill (Nhill Aerodrome 78,031; Queensland Government, 2022).

a Growing Season rainfall is between April and October.

b Day/Month/Year.

c The number of rows (tynes on seeder) sown in each plot and their spacing.

d Mono-Ammonium Phosphate (MAP; Nitrogen: Phosphorus: Potassium: Sulphur: Zinc at 9.2:20.2:0:2.7:2.5).

e 20-year average from 2001 to 2021.

Details of field trial locations, sowing dates, plot sizes, and fertiliser rates are shown in Table 1. Plot sizes varied between experiments depending on the row spacing of the previous cereal crop in the paddock. Rhizobium inoculant (Nodulator® Chickpea granular inoculant, BASF, United States) was sown at 5 kg ha with the seed in each plot. Field sites were maintained free of weeds and insect pests using relevant herbicides and insecticides, respectively.

Each experimental plot (except full control) had 500 ml of AB infested stubble spread evenly across the plot when chickpea plants were at the four-node growth stage (August). From September to November each year, individual plots were visually assessed for disease severity based on the percentage of stems in a plot that were broken because of AB. During 2018, at the Horsham site and 2020 at the Curyo site, rainfall events during pod-fill occurred, resulting in AB pod infection, which were assessed as the percentage of pods with AB lesions present within a plot.

Plots were harvested at crop maturity in December (summer) each season using a small combine harvester with grain yield recorded and a subsample of grain taken for grain quality analysis. Maximum grain yield losses in an experiment as a result of AB were calculated as the difference in yield between full control (T10) to no fungicide (T1) and expressed as a percentage to the yield in the full control treatment.

### Fungicide treatments

#### Horsham and curyo experiments

Eight fungicide strategies were compared to an untreated (no fungicide/diseased, Treatment 1 ‘T1’) and a full control (disease free, T10) strategy at Curyo and Horsham during 2018 to 2020 (Table 2). Seven strategies had Thiram + Thiabendazole (T2-8) and one strategy had a Fluxapyroxad (T9) applied to seed prior to planting (Table 2). Preventative fungicide strategies were applied before rainfall events with single actives [Captan (T2), Chlorothalonil (T3), or Propiconazole (T4)] applied 3–4 weeks apart based on rainfall. This was typical of commercial practice for these products at the time. Recently registered fungicides with different modes of action [Tebuconazole + Azoxystrobin (T5), Bixafen + Prothioconazole (T6)] were also applied in a preventative approach at key growth stages (fourth node and late vegetative/early flowering stage), to maximise foliage protection and ensure a maximum of two applications, as per label directions. Post infection sprays of dual actives [Tebuconazole + Azoxystrobin (T7), Bixafen + Prothioconazole (T8 and T9)] were applied when the first AB lesions were observed and if these first lesions appeared prior to flowering a second application was applied during flowering at least 4 weeks after the first application. Trials were inspected at least weekly for symptoms of AB. Where at least 5 mm of rainfall was forecast within 48 h during podding, Chlorothalonil (1,080 gai/ha) was applied to prevent pod infection, as per the standard industry recommendations.

**Table 2:** Fungicide treatments and the number of applications applied for each treatment to assess control of Ascochyta blight (AB) in chickpea at Curyo and Horsham during 2018–2020.

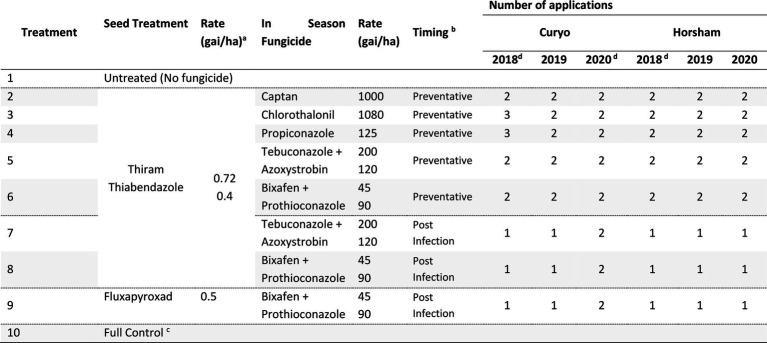

a Grams of active ingredient per hectare.

b Preventative sprays were applied before rainfall events, at key growth stages (fourth node and late vegetative/early flowering stage), to maximise foliage protection. Post infection sprays were applied when the first AB lesions were observed and at flowering. Trials were inspected at least weekly.

c The full control treatment is a rotation of fungicide actives (Chlorothalonil, Tebuconazole + Azoxystrobin, and Bixafen + Prothioconazole) at the rates quoted in the above table applied every 2–3 weeks to ensure minimal to no disease occurred in these plots as a control in the experiment.

d In addition to the fungicides listed an additional podding Chlorothalonil at 1,080 gai/ha was applied to protect seed quality with at least 5 mm of rainfall forecast within 48 h during podding, as per the standard industry recommendations.

#### Nhill experiment

At Nhill, during 2020, five fungicide and one biological control strategies were compared to the full control and no fungicide strategies (Table 3). This experiment compared a recently registered fungicides with multiple modes of action (Fludioxonil + Pydiflumetofen) in Australia and a biological (Trichoderma—BioMAX t4 Primer + BioMAX DigestKicker, Laurie Group, Adelaide, Australia) to Bixafen + Prothioconazole. Fungicide timings were described as either Preventative or Post-infection and applied similarly to the Horsham and Curyo experiments.

**Table 3:** Fungicide treatments and the number of applications applied for each treatment to assess control of Ascochyta blight (AB) in chickpea at Nhill during 2020.

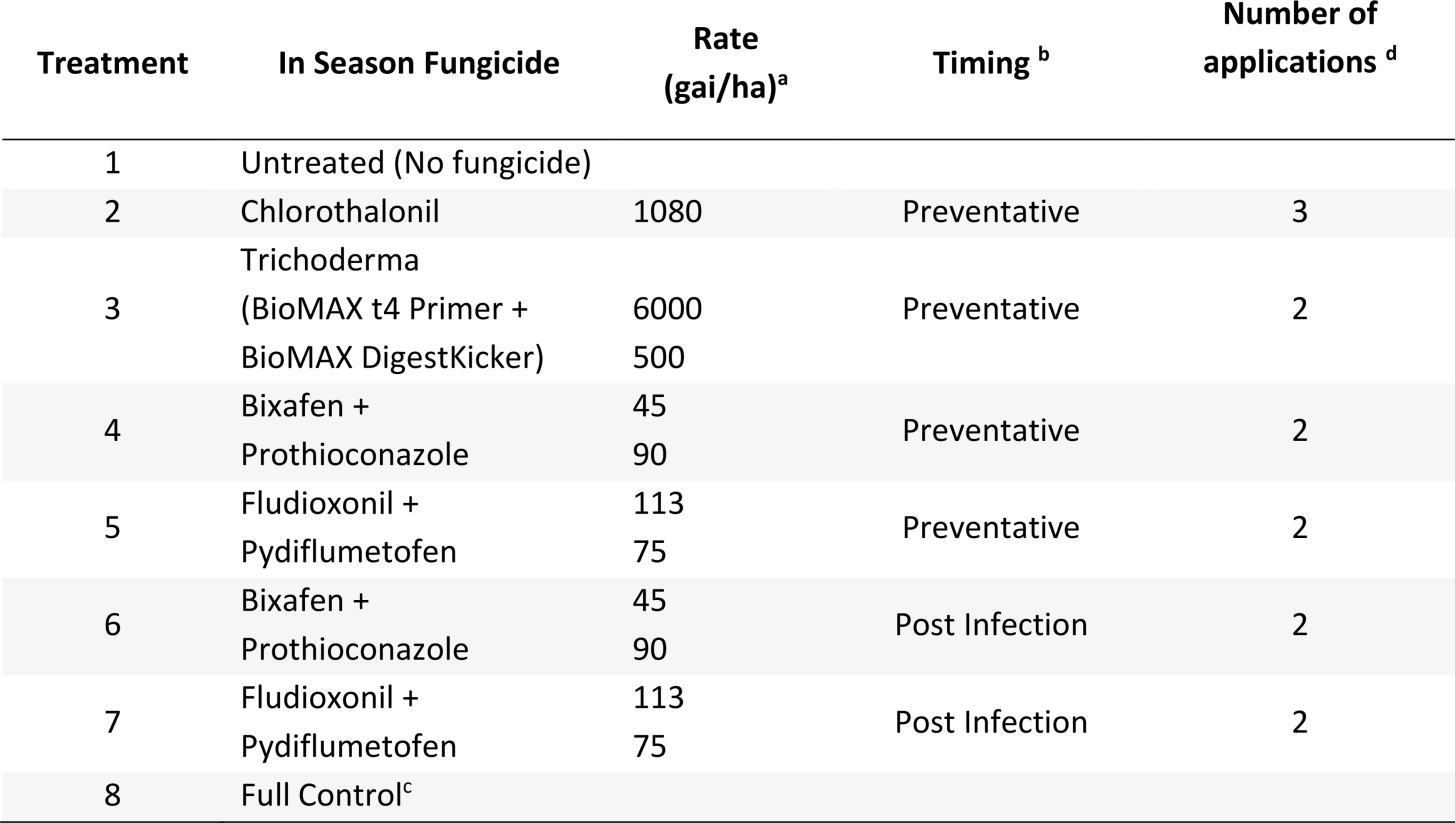

a Grams of active ingredient per hectare.

b Preventative sprays were applied before rainfall events, at key growth stages (fourth node and late vegetative/early flowering stage), to maximise foliage protection. Post infection sprays were applied when the first AB lesions were observed and at flowering. Trials were inspected at least weekly.

c The full control treatment is a rotation of fungicide actives (Chlorothalonil, Tebuconazole + Azoxystrobin, and Bixafen + Prothioconazole) at the rates quoted in the above table applied every 2–3 weeks to ensure minimal to no disease occurred in these plots as a control in the experiment.

d In addition to the fungicides listed an additional podding Chlorothalonil at 1,080 gai/ha was applied to protect seed quality with at least 5 mm of rainfall forecast within 48 h during podding, as per the standard industry recommendations.

### Experimental designs

Each experiment was designed as a split plot with four replicate blocks. Fungicide treatments were randomised to main plots and cultivars to subplots. Designs were produced in Genstat Version 18 (VSNI, United Kingdom). Between each plot (along the 8 m length), a plot of lentils (a non-host) was sown to suppress inoculum movement between plots.

### Grain quality analysis

A random sample of grain (approximately 1,200 seeds) taken at harvest from each plot was scanned through an EyeFoss™ (Foss Analytical, Hoganas, Sweden) to capture multispectral images and concomitant images of grain surface-height, which were processed using MATLAB (The Mathworks Inc., Natick, Massachusetts, United States) software. Seed size characteristics, namely Seed Size Index (SSI, mm) and grain weight (g/100 grains), were computed through image processing according to the methods outlined by LeMasurier et al. (2014) with adaptations for grain volume and seed size distribution. Grain volume was calculated as the sum of voxels in the seed region of each surface height image, and the seed size distribution was adapted to the following size categories: 9, 8, 7, 6, 5, and 4 mm.

### Statistical analysis

Disease severity (%), grain yield (t/ha), grain weight (g), and Seed Size Index (SSI, mm) were analysed using a linear mixed model that accounted for important treatment and cultivar variation as well as extraneous variation associated with design constraints or environmental trends. For each analysis, the model included fixed terms to account for cultivar., fungicide and cultivar by fungicide interaction effects and random terms to account for block and main plot effects. The methodology presented in Gilmour et al. (1997) was used to model the spatial variation in the experiments. If appropriate, linear row and column terms were included in the model to account for possible global trends and random row and column terms for extraneous sources of variation. A separable autoregressive process of order 1 in the column and row direction of the experimental layout was used to model the spatial correlation between neighbouring plots.

Tukey’s tests at 5% significance level were used to assess all the possible pairwise comparisons between means. Where there was no significant interaction between cultivar and fungicide strategy, the two factors act independently to each other and thus the mean response of the cultivar and/or fungicide application is presented (Welham et al., 2014). For the fungicide strategies which were applied under both timing approaches (T5–T8 at Curyo and Horsham and T4–T7 at Nhill), additional contrasts were performed to assess the difference between the preventative vs. the post infection timing. The Wald test at 5% significance level was used to evaluate the significance of the contrasts. The empirical logit transformation was applied to the disease severity data (%) when residuals violated the normality and homoscedasticity assumptions of linear mixed models.

The analyses were conducted in ASReml-R (Butler et al., 2018) in the R statistical software (R Core Team, 2022). Tukey’s tests were performed in the biometryassist R package (Nielsen et al., 2022).

### Gross margin analysis

Gross margins (in Australian dollars) were calculated for all seven experiments to estimate the profits growers could experience from each fungicide strategy for all treatments except the Trichoderma (Nhill T3) strategies which were ineffective and Fluxapyroxad (Curyo and Horsham T9), which was ineffective compared to the Thiram + Thiabendazole seed treatment. Gross margins were calculated by subtracting the cost of the fungicide strategy (chemical (Table 4) and an application cost of $10/ha) from the additional revenue from the increased grain yield compared to the untreated treatment. These fungicide costs were an average of three local (Victoria, Australia) resellers. The grain price ($657/ton) was based on the 10-year (2011–2020) price average (Australian Bureau of Agricultural and Resource Economics and Sciences, 2021).

**Table 4:** Fungicide cost ($/L) and applied cost ($/ha) used in the gross margin analysis.

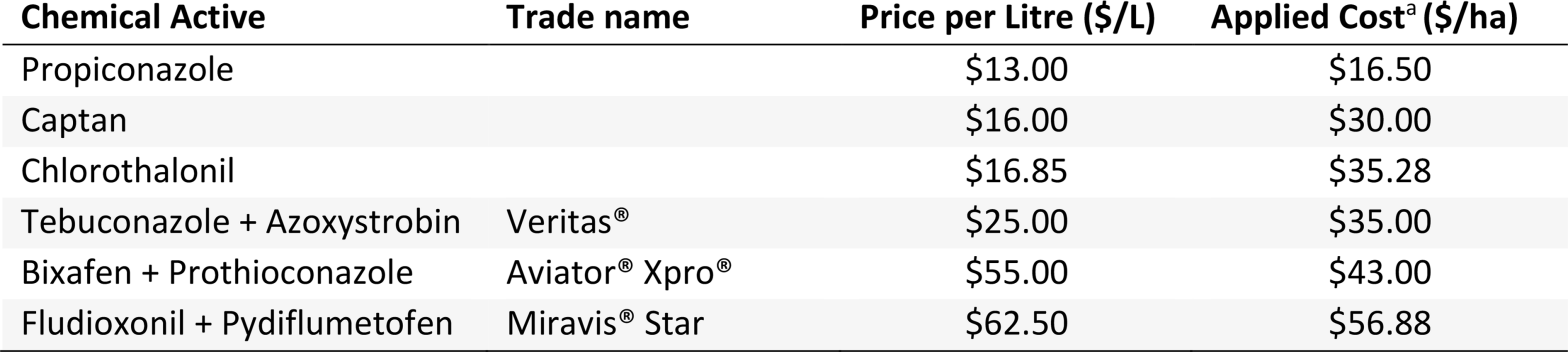

Where there is only one trade name of a fungicide active, these are provided.

a Total cost of chemical (as per rates in Tables 2, 3) plus application ($10/ha).

## Results

### Horsham and curyo experiments

#### Disease severity

There were significant (*p* < 0.05) differences in the disease severities between the fungicide strategies in all experiments and seasons (Tables 5, 6). There was no fungicide strategy that resulted in significantly less disease compared to other strategies across all three seasons. Disease severity varied between seasons in untreated plots with the maximum disease severity at Horsham of 44% during 2018 compared to 87% during 2020. At Curyo, a maximum of 90% disease severity in PBA Striker was observed during 2020 compared to 22% average across cultivars during 2018. In all seasons there was a greater disease severity in the susceptible cultivar PBA Striker as compared to the moderately susceptible cultivar Genesis 090 (Tables 5, 6). A significant interaction between cultivar and fungicide strategy was observed at Horsham during 2020 and at Curyo during 2019 and 2020. Irrespective of the fungicide treatment, disease severity decreased later in the 2020 season at both sites in the cultivar Genesis 090 (Tables 5, 6 and 1Supplementary Data S1, 1S2).

**Table 5:** Chickpea Ascochyta blight disease severity (%) for the significant factors in the fungicide by cultivar experiments at Horsham between 2018 and 2020.

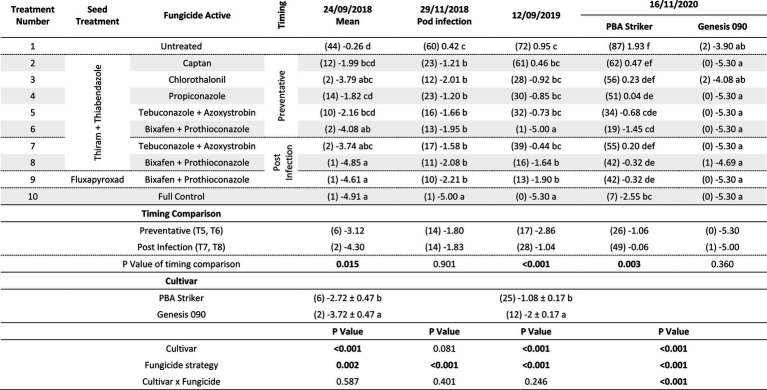

Data are logit transformed with values in parentheses showing back-transformed means. Significant pairwise differences between cultivar or fungicide treatment within an experiment (year) do not have letters in common. Significant values are bolded.

**Table 6:** Chickpea Ascochyta blight disease severity (%) for the significant factors in the fungicide by cultivar experiments at Curyo during 2018 to 2020.

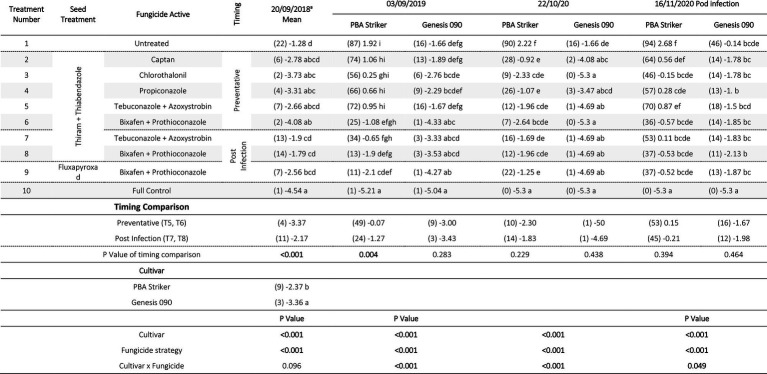

Data are logit transformed with values in parentheses showing back-transformed means. Significant pairwise differences between cultivar or fungicide treatment within an experiment (year) do not have letters in common.

a There was a significant (*p* = 0.008) linear row effect in the Curyo 2018 experiment which was added to the statistical model as a covariate. Significant values are bolded.

A comparison of the post infection fungicide strategies (T7–T8) compared to the preventative fungicide strategies (T5–T6) highlighted a significantly higher disease severity during 2019 and in PBA Striker during 2020 at Horsham and during 2018 at Curyo (Tables 5, 6). However, it was significantly lower at Horsham during 2018 and Curyo in PBA Striker during 2019. Additional disease severity assessments at both sites also highlighted that the difference in disease severity between the post infection fungicide strategies (T7–T8), and the preventative fungicide strategies (T5–T6) were less in the cultivar Genesis 090 compared to PBA Striker (1Supplementary Data S1, 1S2). There was no difference between the Fluxapyroxad (T9) and Thiram + Thiabendazole (T8) seed treatments on disease severity with the post-infection fungicide strategy (Tables 5, 6).

#### Grain yield

In all experiments, except for 2018 at Curyo there was a significant (*p* < 0.005) fungicide strategy treatment effect (Tables 7, 8). During 2018 at Curyo, there were no significant differences in grain yield. However, the cultivar PBA Striker had a trend (*p* = 0.053) toward a lower grain yield compared to Genesis 090 with grain yields of 0.44 ± 0.01 and 0.48 ± 0.01 t/ha, respectively. Grain yield losses varied from 38% (0.98 t/ha) in Genesis 090 at Horsham during 2020 to 96% (2.44 t/ha) yield loss in PBA Striker at Curyo during 2020.

**Table 7:** Chickpea grain yields (t/ha) in the presence of Ascochyta blight for the significant factors in the fungicide by cultivar experiments at Horsham between 2018 and 2020.

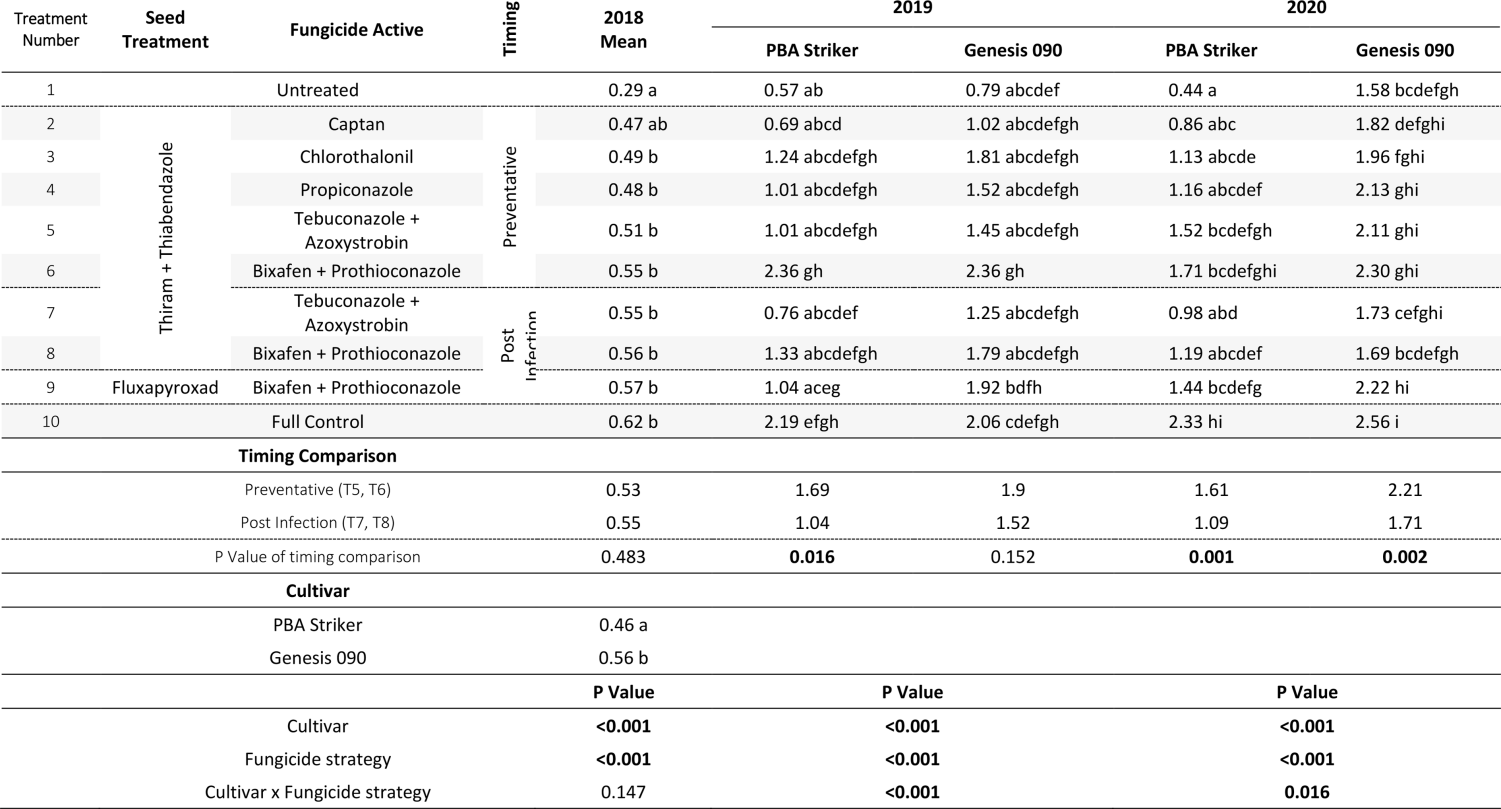

Significant pairwise differences between cultivar or fungicide treatment within an experiment (year) do not have letters in common. Significant values are bolded.

**Table 8:** Chickpea grain yields (t/ha) in the presence of Ascochyta blight for the significant factors in the fungicide by cultivar experiments at Curyo between 2019 and 2020.

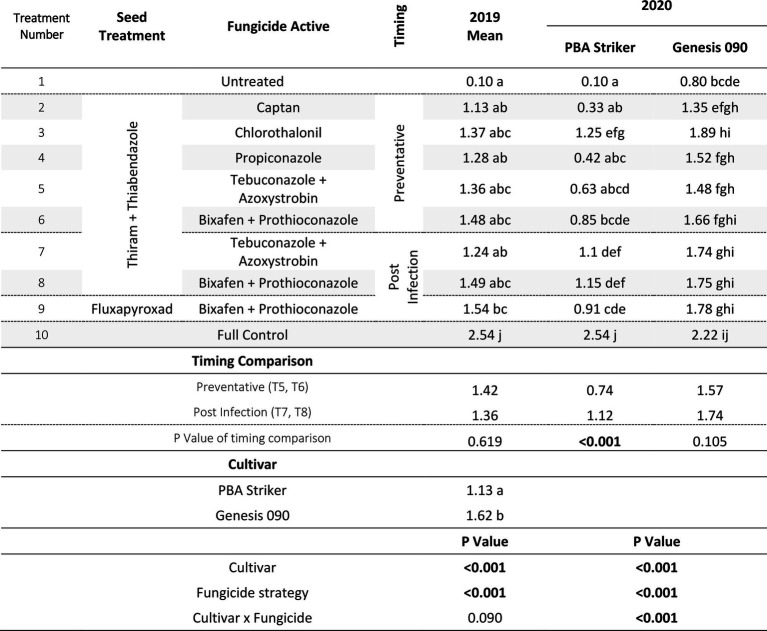

Significant pairwise differences between cultivar or fungicide treatment within an experiment (year) do not have letters in common. Significant values are bolded.

In the absence of fungicides, the cultivar Genesis 090 consistently yielded significantly higher than PBA Striker (Tables 7, 8), except at Horsham during 2019. In the full fungicide control, in the experiments where there was a significant cultivar × fungicide strategy interaction there was no significant difference in yield potential of the two cultivars (Tables 7, 8).

All fungicide strategies increased grain yield except in the 2018 Curyo experiment (Tables 7 and 8). Within the dual active products Bixafen + Prothioconazole (T6–T9) had a trend to yield consistently higher than Tebuconazole + Azoxystrobin (T5 and T7), although not significant in most experiments. There was no difference between the Fluxapyroxad (T9) and Thiram + Thiabendazole (T8) seed treatments on grain yield with the post-infection fungicide strategy (Tables 7, 8).

A direct comparison in the post infection fungicide strategies (T7–T8) compared to the preventative fungicide strategies (T5–T6) showed a 0.65 t/ha yield loss in PBA Striker at Horsham during 2019 and 0.5–0.52 t/ha grain yield loss in PBA Striker and Genesis 090, respectively, during 2020 (Table 7). At Curyo, there was a 0.38 t/ha yield increase during 2020 in PBA Striker (Table 8).

#### Grain quality

The cultivar PBA Striker had a significantly lower grain weight than Genesis 090 during 2018 to 2020 (Tables 9, 10). During 2020 at Curyo there was a greater grain weight in the full control plots as compared to untreated plots, which was also observed during 2020 at Horsham in the cultivar Genesis 090 (Table 10). The 2019 Horsham grain was not analysed due to mould in the grain after harvest. A smaller seed size index (SSI) was also associated with the untreated plots as compared to the full control, although not significant and the desi chickpea PBA Striker had a significantly smaller SSI as compared to the Kabuli chickpea Genesis 090 (1Supplementary Data S3). The timing approach of preventative or post infection fungicides did not seem to have an effect on grain quality (Table 10, 1Supplementary Data S3).

**Table 9:** Grain weight (g/100 grains) differences between cultivars in the fungicide by cultivar experiments at Curyo in 2018 and 2019 and Horsham in 2018.

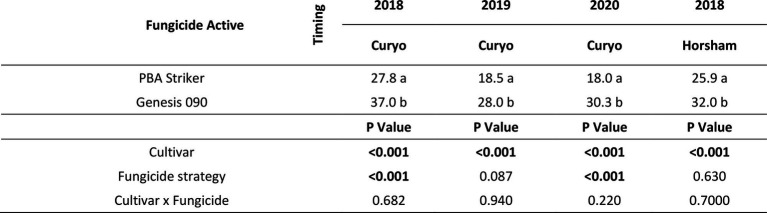

Significant pairwise differences between cultivar or fungicide treatment within an experiment (year) do not have letters in common. Significant values are bolded.

**Table 10:** Chickpea grain weight (g/100 grains) in the presence of Ascochyta blight for the significant factors in the fungicide by cultivar experiments at Curyo during 2020 and Horsham during 2020.

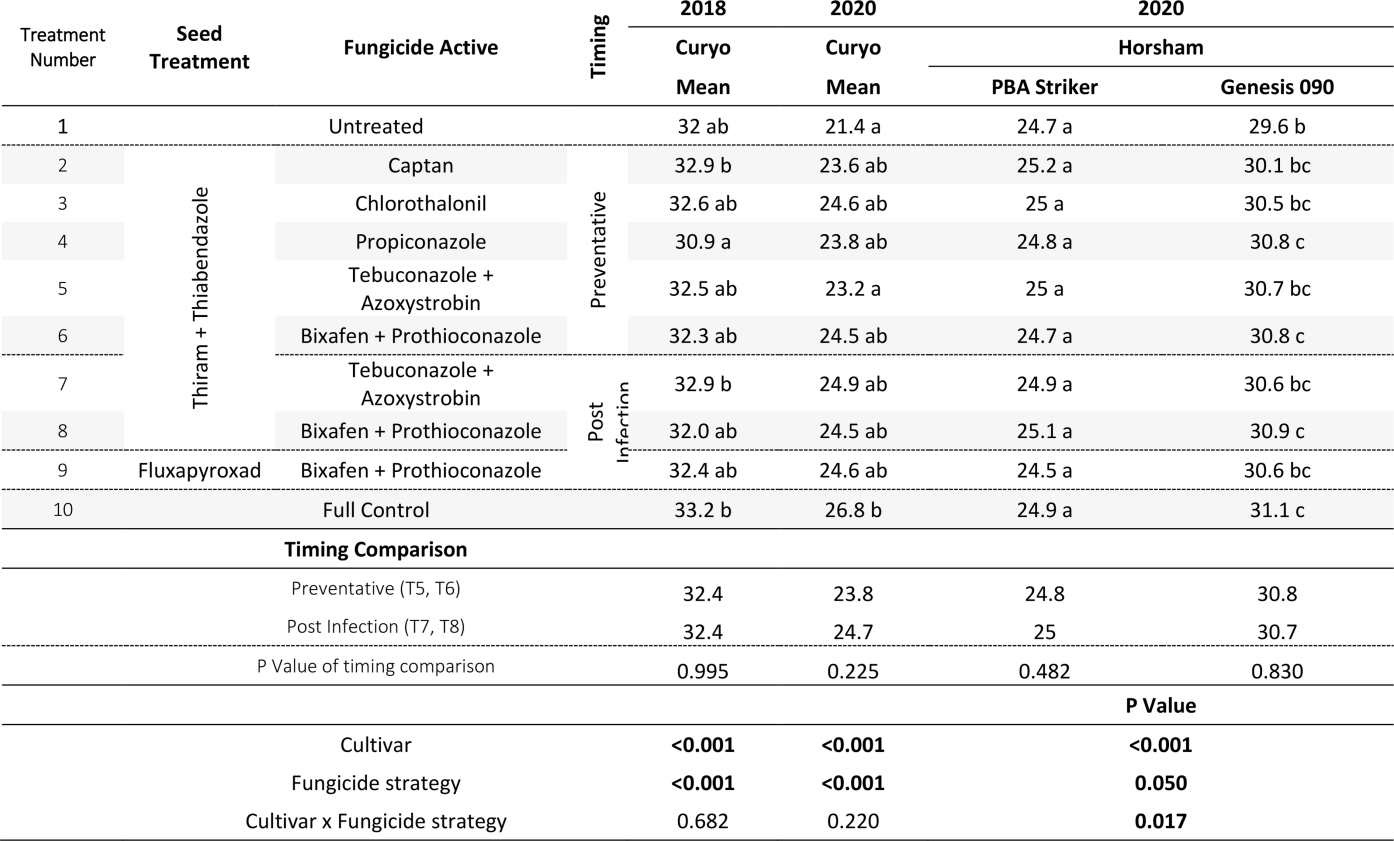

Significant values are bolded.

#### Gross margins

The gross margin results varied considerably between fungicide strategies, cultivars, and experiments and there was no single fungicide strategy that consistently had a higher gross margin. The highest gross margin observed was in a dual active product Bixafen + Prothioconazole applied to PBA Striker preventative (T6), resulting in $1,095/ha at Horsham during 2019 (Table 11). Gross margins of the tested fungicide strategies were positive except for Curyo during 2018 (Tables 11, 12). In this experiment, economic losses were lower using the post-infection strategies (T7–T9) or Propiconazole (T4) strategies compared to the other strategies (Table 12). Similarly, at Horsham during 2018 and Curyo during 2019, the post infection fungicide strategies (T7 and T8) were more profitable as compared to the same fungicides applied preventative (T5 and T6). However, in all other seasons, the preventative timings using the same fungicides were more profitable than the post-infection application.

**Table 11:** Gross Margin ($AUD/ha) as compared to the untreated control at Horsham between seven fungicide strategies to control Ascochyta blight and two cultivars with differing resistance, between 2018 and 2020.

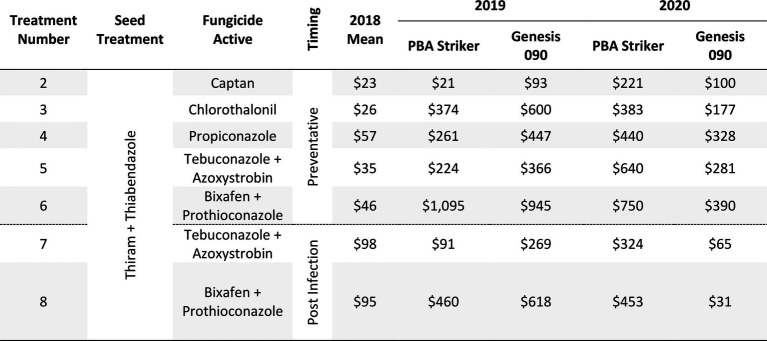

**Table 12:** Gross Margin ($AUD/ha) at Curyo between seven fungicide strategies to control Ascochyta blight and two cultivars with differing resistance, between 2018 and 2020.

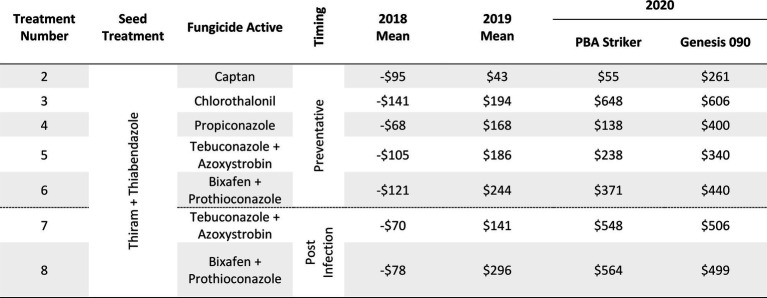

### Nhill experiment

#### Disease severity

In Nhill, during 2020, the disease severity ranged from 1 to 87%. The lowest disease severity was in all Genesis 090 plots and the PBA Striker full control plots (Table 13). This low disease severity in Genesis 090 in all fungicide treatments was not at every point through the season as plants recovered later in the season (1Supplementary Data S4). In PBA Striker, the post infection Bixafen + Prothioconazole (T6) was not significantly different compared to the untreated control plots (T1), and the preventative timing (T4) had significantly lower disease severity in comparison to the untreated control plots (T1). In PBA Striker, the post infection applications (T6 and T7) had 30% higher disease severity as compared to the preventative fungicide applications (T4 and T5). Two out of the five disease severity assessments highlighted a greater disease severity in the post infection fungicide timings compared to the preventative fungicide timing in both cultivars (Table 13, 1Supplementary Data S4). However, in the cultivar Genesis 090, the three later (after 23/09/20) disease severity assessments highlighted no significant difference between the preventative and post infection timings (Table 13, 1Supplementary Data S4).

**Table 13:** Chickpea Ascochyta blight disease severity (% on 16/11/2020), grain yields (t/ha) and Grain weight (g/100 grains), at Nhill in two cultivars of chickpea treated with 10 different fungicide strategies.

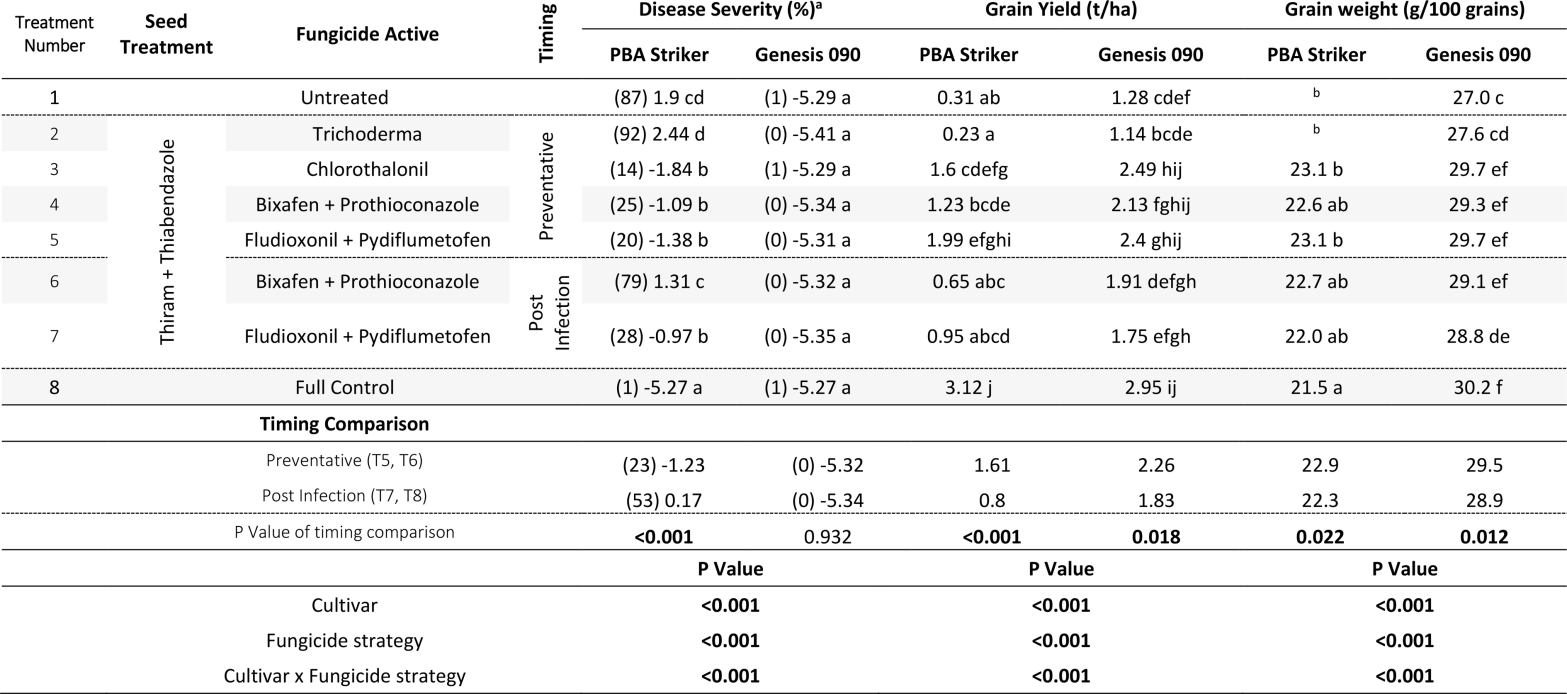

a Data is logit transformed with values in parentheses showing back-transformed means.

Significant pairwise differences between cultivar or fungicide treatment within an experiment (year) do not have letters in common There was a significant (*p* = 0.039) linear row effect in the disease severity data which was added to the statistical model as a covariate.

b The grain yield was too small to analyse grain weight. Significant values are bolded.

#### Grain yield

There was a significant (*p* < 0.05) interaction between the cultivar × fungicide strategy (Table 13). There was a significantly lower grain yield in the untreated PBA Striker plots as compared to the untreated Genesis 090 plots. Although not significant for the Bixafen + Prothioconazole (T4 vs. T6) there was also a trend towards a lower grain yield in the post infection fungicide timing (T6) as compared to the preventative timings of the same fungicides (T4). This was significant for Fludioxonil + Pydiflumetofen, with greater grain yield observed in the preventative timing (T5) compared to the post infection (T7) timing in PBA Striker. Comparing the means of the preventative applications (T4 and T5) compared to the post infection (T6 and T7) fungicide applications, there was a 0.81 and 0.43 t/ha grain yield increase in the preventative strategies in PBA Striker and Genesis 090, respectively. The Trichoderma (T2) strategy in both cultivars did not result in significantly increased grain yield as compared to the untreated control (T1).

#### Grain quality

There was a highly significant (*p* < 0.005) cultivar × fungicide strategy effect on 100 grain weight (Table 13) and cultivar and treatment effect on SSI (1Supplementary Data S3). A greater grain weight and SSI was observed in Genesis 090 as compared to PBA Striker. The fungicide strategy effects were very small (Tables 13 and 1Supplementary Data S3). However, there was a significantly lower grain weight associated with the post infection fungicide applications as compared to the preventative fungicide applications (Table 13). The untreated and Trichoderma strategies had a harvest too small to analyse grain quality. In Genesis 090, a lower 100 grain weight was observed in the plots which had a lower grain yield.

#### Gross margins

The gross margin results varied considerably between fungicide strategies and cultivars (Table 14). The highest gross margins were observed in the preventative timing (T4 and T5) compared to the post infection timing (T6 and T7; Table 14). The Chlorothalonil applied preventative (T3) also had higher gross margins than the dual actives applied post infection (T6 and T7). The highest gross margins were observed in the preventative applications of Fludioxonil + Pydiflumetofen (T5).

**Table 14:** Gross Margin ($AUD/ha) at Nhill between five fungicide strategies to control Ascochyta blight and two cultivars with differing resistance, during 2020.

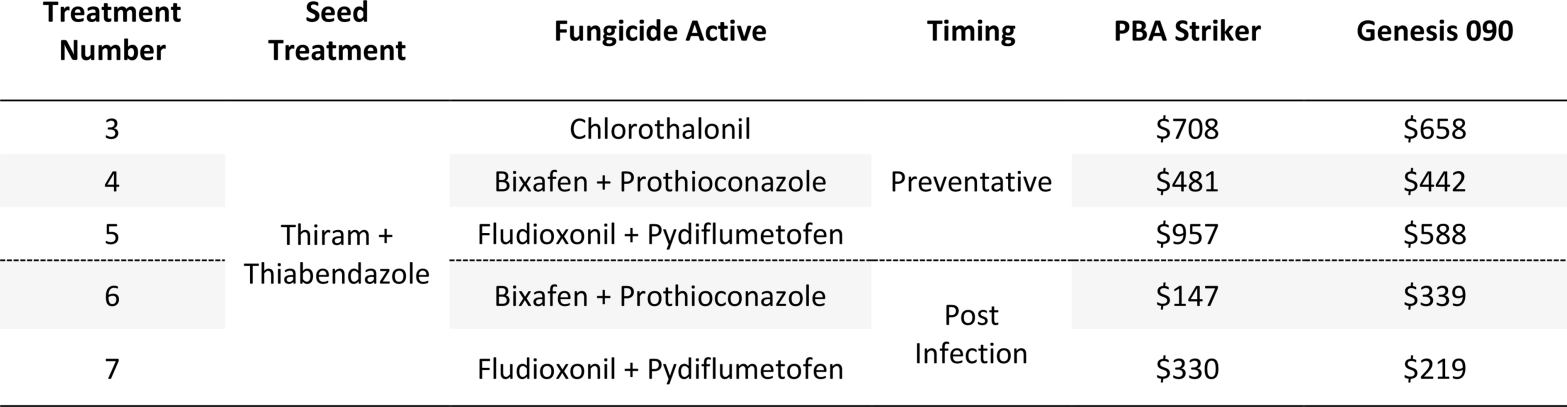

## Discussion

Grain yield losses due to AB in Victoria Australia were shown to be as high as 96% or 2.44 t/ha in the susceptible cultivar PBA Striker, at Curyo during 2020, highlighting the importance of implementing control measures. Demonstrating the benefit of cultivar resistance, in the same experiment at Curyo during 2020, the moderately susceptible cultivar., Genesis 090, had only 64% or 1.41 t/ha grain yield loss. These high grain yield losses were because of high disease severity with up to 90 and 16% stem breakage observed in PBA Striker and Genesis 090, respectively, during October in this experiment. These grain yield losses were similar to those previously reported in susceptible cultivars in Australia 10 years ago (Bretag et al., 2008). In contrast to Bretag et al. (2008) who reported a 4% grain yield loss in the cultivar Genesis 090, this study showed up to 64% yield loss at Curyo during 2020, highlighting the difficulty in finding durable AB genetic resistance, with AB overcoming new cultivar resistance sources. In the same 2020 Curyo experiment, Genesis 090 had grown away from the disease in this season with a greater disease severity observed earlier in the season at 68% compared to later in the season. This highlights that although plants can appear to recover and show a level of resistance, grain yield loss may still have occurred due to the stem breakage earlier in the season.

Maximum grain yields recorded across seasons were 2.56 t/ha in Horsham, 2.54 t/ha in Curyo, and 3.12 t/ha in Nhill. Despite the variation in rainfall between sites, the maximum grain yield varied only 0.58 t/ha between sites across the different production zones, highlighting a similar yield potential between the environments. Despite small variations in the maximum grain yields, the efficacies of fungicides varied between experiments and seasons and thus the gross margins also varied. The greatest gross margins were observed in the higher rainfall environments and seasons where fungicides with multiple modes of action were applied preventatively. This is similar to results presented by Bretag et al. (2008) where they found greater gross margins with higher disease pressure and higher rate of fungicides.

In more conducive seasons, fungicide strategies did not fully prevent AB, with even the full control at Horsham during 2020 having low (7%) levels of disease observed in PBA Striker. However, in the moderately susceptible cultivar Genesis 090, disease severity was significantly lower, and the fungicides were much more effective at reducing disease severities. This interaction between cultivar and season has been previously reported in other studies showing that it is difficult to control AB in higher rainfall seasons with susceptible cultivars (Demirci et al., 2003; Bretag et al., 2008). The results showed that the recently registered fungicides with dual actives being tested in this study were still not able to completely control AB, which highlights the need for resistant cultivars to be used in combination with other control strategies.

The comparison of fungicide applications with multiple modes of action, applying fungicides before a rainfall event (preventative) compared to after the first signs of disease (post-infection) highlighted no clear advantage to either approach at Curyo in both grain yield and disease severity. At Horsham and Nhill, there seemed to be a clear advantage of the preventative fungicide timings as compared to the post infection timings. This message was clearer in the cultivar PBA Striker compared to Genesis 090, where lower grain yield and higher disease severity was usually (except 2018) recorded in PBA Striker. During 2018 when there was a yield potential year of <0.62 t/ha, the economic losses were reduced at Curyo and there was a higher profitability of the post infection applications at Horsham. All variables (disease severity, grain yield, and profitability) varied considerably between experiments and seasons. Post infection fungicide approaches may prove to be profitable for growers in lower rainfall seasons or environments where AB is not observed in every season, and there is the ability to apply fungicides reactively in a timely manner on observation of disease development. This approach would be a higher risk disease strategy, as there is a chance disease severity could increase rapidly if post infection fungicides are not applied in a timely manner. However, in combination with partially resistant cultivars, the post infection fungicide applications may reduce growers’ economic losses when seasonal variability is an issue. In higher rainfall seasons and years, the proactive high-cost fungicide strategies appeared most profitable, likely due to high disease pressure with the maximum gross margin of $1,095/ha observed during 2019 applying Bixafen + Prothioconazole to PBA Striker.

One experiment at Nhill during 2020 tested a biological (Trichoderma) management strategy. Trichoderma products have been utilised by growers on the premise of AB management locally in Victoria with mixed results and this study aimed to compare them to synthetic fungicides. Previously, Poveda (2021) has reported that several *Trichoderma* spp. reduced AB infection *in vitro* and in glasshouse conditions. This study, with a single experiment utilising Trichoderma, found no significant differences between the untreated control and the *Trichoderma* treatment applied in a similar method to the synthetic fungicides. Further research into the application of *Trichoderma* in a broad acre field situation is required before any definitive recommendation would be provided to industry on the use of Trichoderma as an AB management strategy.

A higher grain weight and higher seed size index were observed in the full control treatment as compared to no fungicide. The higher grain weights were also the plots with a lower AB disease severity, indicating that an increase in AB disease severity resulted in a decrease in grain weight. Previous studies have shown variable results on grain weight with some reporting no differences and others small differences (Iqbal et al., 2003; Banniza et al., 2011). In most experiments, a higher grain weight was associated with the cultivar Genesis 090 as compared to PBA Striker, which is expected with Genesis 090 a Kabuli type and PBA Striker a desi type (Hossain et al., 2010). Further investigations would be required to verify results and the differences observed here, which although significant, were only minor in most experiments except the Curyo 2020 experiment where AB pod infection was observed.

The profitability between fungicides was highly variable, but all fungicides in most seasons provided an economic advantage, which gives confidence to growers to rotate fungicides and still be profitable while reducing the chance of fungicide resistance developing. This includes all the fungicides tested, including the preventative use of single active ingredient fungicides of Captan, Chlorothalonil, and Propiconazole will protect chickpea from AB and improve profitability in most seasons. Although Chlorothalonil is a multisite fungicide with very rare chance of resistance developing it is widely used and can have over four applications per season on susceptible cultivars, which will increase the risk of resistance developing (Bretag et al., 2008; Corkley et al., 2022; Crutcher et al., 2022). The effectiveness of all fungicides allows growers the opportunity to rotate fungicides, reducing the chance of fungicide resistant pathogen populations developing.

The results in this study have shown: (1) the importance of cultivar resistance in reducing AB disease severity and subsequent grain yield losses, (2) the fungicide strategies tested are profitable in controlling AB in most seasons, and (3) a post infection fungicide in some environments is effective and profitable. Therefore, this study provides growers with more disease management strategies to prolong the life of the currently available fungicides, strategies to reduce grain yield losses due to AB, and strategies to improve on farm profitability.

The benefit of cultivar resistance clearly highlights the importance of continued research into improving resistance. For dual active fungicides applied under the two application approaches evaluated in this study, the preventative timing is recommended for susceptible cultivars. Once cultivars with improved resistance are available to growers, the effectiveness of the preventative vs. post infection fungicide applications should be further investigated as differences between both approaches may be reduced.

## Data availability statement

The original contributions presented in the study are included in the article/Supplementary material; further inquiries can be directed to the corresponding author.

## Author contributions

JF and JB designed and implemented the experiments and completed all assessments with technical assistance. IM and JT completed all data analysis. JF wrote the article and led the manuscript process with significant assistance from GH, IM, JB, LM, and JT. All authors contributed to the article and approved the submitted version.

## Funding

Co-investment for this research to occur was received from the Grains Research and Development Corporation (GRDC) and the Victorian Government (DAV00150 and DJP1907-001RTX).

## Conflict of interest

This study received funding from Grains Research and Development Corporation (GRDC). The funder had the following involvement with the study: Two investment managers proofread the manuscript prior to submission making minor spelling and grammar corrections. All authors declare no other competing interests.

## Publisher’s note

All claims expressed in this article are solely those of the authors and do not necessarily represent those of their affiliated organizations, or those of the publisher, the editors and the reviewers. Any product that may be evaluated in this article, or claim that may be made by its manufacturer, is not guaranteed or endorsed by the publisher.
